# miR‐92b promotes gastric cancer growth by activating the DAB2IP‐mediated PI3K/AKT signalling pathway

**DOI:** 10.1111/cpr.12630

**Published:** 2019-11-12

**Authors:** Qing‐feng Ni, Yan Zhang, Jia‐wei Yu, Ru‐heng Hua, Qu‐hui Wang, Jian‐wei Zhu

**Affiliations:** ^1^ Department of General Surgery Affiliated Hospital of Nantong University Nantong China; ^2^ Department of Chemotherapy Affiliated Hospital of Nantong University Nantong China

**Keywords:** DAB2IP, gastric cancer, miR‐92b, PI3K/AKT pathway, proliferation

## Abstract

**Objectives:**

miR‐92b has been reported to play critical roles in several carcinomas; however, our understanding of the mechanisms by which miR‐92b stimulates gastric cancer (GC) is incomplete. The aim of this study was to investigate the clinical significance and functional relevance of miR‐92b in GC.

**Materials and methods:**

Expression of miR‐92b in GC and peritumoural tissues was determined using qRT‐PCR, in situ hybridization and bioinformatics. CCK‐8, colony formation and fluorescence‐activated cell sorting assays were utilized to explore the effect of miR‐92b on GC cells. A luciferase reporter assay and Western blotting were employed to verify miR‐92b targeting of DAB2IP. Furthermore, Western blotting was used to evaluate the levels of DAB2IP and PI3K/Akt signalling pathway‐related proteins.

**Results:**

In this study, we found that miR‐92b was upregulated in GC tissues compared with peritumoural tissues. Overexpression of miR‐92b promoted cell proliferation, colony formation, and G_0_/G_1_ transition and decreased apoptosis. Our results indicated that miR‐92b repressed the expression of DAB2IP and that loss of DAB2IP activated the PI3K/AKT signalling pathway. Overexpression of DAB2IP rescued the effects of miR‐92b in GC cells. Finally, our results demonstrated a significant correlation between miR‐92b expression and DAB2IP expression in GC tissues.

**Conclusions:**

Our results suggest that miR‐92b promotes GC cell proliferation by activating the DAB2IP‐mediated PI3K/AKT signalling pathway. The miR‐92b/DAB2IP/PI3K/AKT signalling axis may be a potential therapeutic target to prevent GC progression.

## 1 INTRODUCTION

Gastric cancer (GC) is currently the most common malignancy of the gastrointestinal tract, with a high mortality rate that ranks as the second most diagnosed cause of cancer‐related death worldwide.^1^ According to recent statistics, approximately one million newly diagnosed cases of GC are reported annually, and in 2013, the number of GC‐related deaths was nearly 723 000.^2^, ^3^ Surgical resection remains the primary treatment for GC patients and can provide the best chance of cure, and chemotherapy and radiotherapy for GC can inhibit tumour cell growth or invasion.^4^, ^5^ Despite multiple treatment interventions for GC patients, their 5‐year survival rate remains pessimistic due to recurrence and metastasis.^6^ Hence, in view of its poor outcomes, GC development and recurrence must be prevented, and effective early diagnostic biomarkers and therapeutic targets must be identified to improve GC patient prognosis.

MicroRNAs (miRNAs), a class of small and non‐coding RNAs (19‐22 nucleotides in length), are well‐known master regulators of the human genome that regulate genes transcriptionally or post‐transcriptionally by binding with the 3′ untranslated region (3′‐UTR) of target messenger RNA (mRNA) in a sequence‐specific manner, thereby decreasing or increasing gene expression.^7^ Accumulating evidence has firmly established specific miRNAs as targets in genomic lesions that functionally contribute to activation or inactivation of oncogenes or tumour suppressors in many different human cancers, including GC.^8^, ^9^

miR‐92b, which has various functions, was reported to be induced by PPAR‐γ and to inhibit Axl expression, in turn reducing the expression of TGF‐β1 and downstream genes in keloid fibroblasts and attenuating oncogenicity effects by directly targeting Smad3, RAB23 and Gabra3 in nasopharyngeal cancer, oesophageal squamous cell carcinoma and pancreatic cancer.^10^, ^11^, ^12^, ^13^ These studies indicated that miR‐92b might act as a tumour suppressor in some human cancers; however, miR‐92b also might have different functions in different tumours. It has been reported that miR‐92b promotes non‐small‐cell lung cancer cell growth and motility by targeting RECK.^14^ Song et al^15^ showed that enhanced miR‐92b expression increases glioma cell proliferation, metastasis and apoptosis via the PTEN/AKT signalling pathway. However, the effect of miR‐92b on cancer is still uncertain, and the correlation between miR‐92b and GC remains unknown. Whether miR‐92b can influence oncobiological functions and be applied as an efficient or specific anti‐cancer therapeutic in GC needs to be investigated.

DAB2 interactive protein (DAB2IP), a Ras GTPase‐activating protein family member, is a well‐known tumour suppressor gene in a variety of human cancers.^16^ DAB2IP has been found to suppress PI3K‐AKT signalling and inhibit cell survival and proliferation in prostate cancer.^17^ The role of the PI3K‐AKT pathway in malignant diseases has been widely studied, and altered expression or mutation of many components of this pathway has been implicated in human cancer.^18^, ^19^, ^20^ In our study, we discovered that DAB2IP a target of miR‐92b and that the DAB2IP‐mediated PI3K‐AKT signalling pathway is activated in GC.

## 2 MATERIALS AND METHODS

### 2.1 Patients and tissue collection

The Human Research Ethics Committee of Affiliated Hospital of Nantong University, China, approved this project. Signed informed consent was obtained from all subjects, and no scientific research was conducted without informed consent. GC tissues and paired normal adjacent mucosa tissues were derived from 59 GC patients who had undergone surgery at the Affiliated Hospital of Nantong University between 2012 and 2013. Regular post‐operative follow‐up was arranged and lasted until June 2017. None of the subjects recruited for this project received any therapy prior to surgery. Surgical specimens were diagnosed by pathological examination. Histological classifications and TNM stages were determined independently by two senior pathologists according to the classification criteria of the American Joint Committee on Cancer. Resected samples were snap‐frozen in liquid nitrogen until RNA and protein extraction could be performed.

### 2.2 Cell lines and cell culture

Five human GC cell lines (SGC7901, MGC803, AGS, MKN45 and BGC823) and a human normal gastric epithelial cell line (GES‐1) were purchased from American Type Culture Collection and National Infrastructure of Cell Line Resource. The cell lines were maintained in RPMI 1640 medium supplemented with 10% foetal bovine serum, ampicillin and streptomycin in a humidified chamber at 37°C with 5% CO_2_.

### 2.3 RNA isolation and quantitative real‐time polymerase chain reaction

Total RNA isolation and quantitative real‐time polymerase chain reaction (qRT‐PCR) analyses of miR‐92b and DAB2IP expression were performed as previously reported.^21^ The relative expression levels of miR‐92b and DAB2IP were calculated in human tissues and cultured cell lines using the comparative *C*_t_ method.^22^ The miR‐92b expression level was normalized to that of U6, and the expression of DAB2IP was normalized to that of GAPDH. The sequences of the primers are listed in Table 1.

**Table 1:** The nucleotides applied in the study

Description	Name	Sequence	
Primers for qRT‐PCR	hsa‐miR‐92b forward		5′‐GGGGCAGTTATTGCACTTGTC‐3′
	hsa‐miR‐92b reverse		5′‐CCAGTGCAGGGTCCGAGGTA‐3′
	U6 forward		5′‐CTCGCTTCGGCAGCACA‐3′
	U6 reverse		5′‐AACGCTTCACGAATTTGCGT‐3′
	DAB2IP forward		5′‐TGGACGATGTGCTCTATGCC‐3′
	DAB2IP reverse		5′‐GGATGGTGATGGTTTGGTAG‐3′
	GAPDH forward		5′‐ATGGGGAAGGTGAAGGTCGG‐3′
	GAPDH reverse		5′‐GACGGTGCCATGGAATTTGC‐3′
Primers for in situ hybridization	Locked nucleic acid		5′‐UAUUGCACUCGUCCCGGCCUCC‐3′
Plasmid construction for DAB2IP	DAB2IP forward		5′‐AGAGATATCATGGAGCCCGACTCCCTTCT‐3′
	DAB2IP reverse		5′‐AGAGAATTCTAATGCATACTCTCTTTCAGCTGGG‐3′
Sequence of siRNA‐1309	Sense		5′‐GACGCCCUAGGUGAGUUCAUCAAAGdTdT‐3′
	Anti‐sense		5′‐CUUUGAUGAACUCACCUAGGGCGUC dTdT‐3′

### 2.4 Protein extraction and Western blotting

Protein isolation and Western blotting analysis were performed as previously reported.^23^ Antibodies against DAB2IP, cyclin D1, p21, p27, Bax, Bcl‐xl, Bcl‐2, PI3K, p‐PI3K, AKT, p‐AKT and GAPDH were purchased from Abcam. GAPDH was employed as the loading control.

### 2.5 Immunohistochemical staining and in situ hybridization

Gastric cancer tissues, adjacent normal tissues and nude mouse subcutaneous tumours were fixed with 4% paraformaldehyde and embedded in paraffin. The methods for immunohistochemical staining (IHC) and in situ hybridization (ISH) were conducted and evaluated as previously described.^24^ ISH analysis was conducted employing an anti‐sense locked nucleic acid (LNA)‐modified probe on the basis of the sequence of hsa‐miR‐92b (Boster). The detailed sequence of the LNA is presented in Table 1. The immunoreactive score (IRS) for the proportion of positive cells and the staining grade were calculated for each specimen. The quantity score evaluated the proportion of cells positive for miR‐92b, DAB2IP or ki67 and was determined as follows: 1 (0%‐25% labelled cells), 2 (26‐50 labelled cells), 3 (51%‐75% labelled cells) or 4 (76%‐100% labelled cells). The intensity score evaluated the intensity of staining and was scored as 0 (negative staining), 1 (weakly positive), 2 (moderately positive) or 3 (strongly positive). Staining extent and intensity scores were multiplied to calculate an IRS ranging from 0 to 12: an IRS > 3 was defined as high miR‐92b or DAB2IP expression, and an IRS ≤ 3 was defined as low miR‐92b or DAB2IP expression.

### 2.6 Transfections and plasmid construction

Lentiviral vectors encoding miR‐92b mimics, miR‐92b inhibitor or the controls were constructed according to the manufacturer's protocol (GenePharma), and the constructed vectors were verified by DNA sequencing and then used to transfect GC cells to induce miR‐92b overexpression or knock‐down. The plasmid for DAB2IP was created using pcDNA3.1 (Invitrogen). Human genomic DNA was used as a template for PCR amplification, and the DAB2IP open reading frame (GenBank: NM_032552.3) was subcloned into the pcDNA3.1 expression vector. A synthesized DNA nucleotide fragment encoding shRNA against endogenous DAB2IP was designed according to Invitrogen (Thermo Fisher Scientific, Inc) and synthesized by GenePharma Co., Ltd. The sequences were incorporated into the vector p‐SUPER (OligoEngine) to generate p‐SUPER‐sh‐RNA. The detailed primer and siRNA sequences are listed in Table 1. The transfection process was performed as previously reported.^25^

### 2.7 Cell proliferation assay

Transfected cells were seeded at a density of 2000 cells per well in 96‐well plates at daily intervals (every 24 hours for 5 days) using a Cell Counting Kit 8 (Dojindo) according to the manufacturer's protocol. The absorbance measured at 450 nm was employed to evaluate the growth curve of transfected cells.

### 2.8 Colony formation assay

Each type of the SGC7901 and BGC823 cells at a density of 500 cells per well was seeded into 6‐well plates and then maintained for 2 weeks in medium containing G418 (1000 µg/mL). Paraformaldehyde was employed to fix proliferating colonies, which were then stained with crystal violet. After the stained colonies were photographed under a microscope (Leica), the number of colonies (>50 cells/colony) was determined.

### 2.9 Flow cytometric analysis of cell cycle

The cell cycle assay was performed as previously reported.^26^ The proportion of cells in G_0_/G_1_, S and G_2_/M cell cycle phases was evaluated by employing Cell Quest acquisition software (BD Biosciences).

### 2.10 Flow cytometric analysis of cell apoptosis

Fluorescence‐activated cell sorting (FACS) was used to detect cell apoptosis. The induction and cell apoptosis measurement methods were performed as previously reported.^27^

### 2.11 Subcutaneous tumour growth assay

BALB/c nude male mice, 4 weeks old, were purchased from the Department of Laboratory Animal Center, Nantong University. Animal experiments were performed according to a protocol approved by the Nantong University Ethics Committee. A total of 100 µl PBS mixed with 1 × 10^6^ control or manipulated cells was subcutaneously inoculated into each flank of the nude mice. Tumour size in the inoculated nude mice was monitored and measured every third day. The mice were sacrificed, and the subcutaneous tumours were dissected out and weighed after 4 weeks. Tumour volume was calculated from the following formula: tumour volume = 1/2 × length × width^2^_. _The removed tumours were flash frozen and stored in liquid nitrogen in preparation for further studies.

### 2.12 Luciferase reporter assay

The underlying binding site of miR‐92b in the 3′‐UTR of DAB2IP mRNA was predicted by the TargetScan, PicTar and miRanda databases. The 3′‐UTR sequences of DAB2IP containing miR‐92b binding sites (wild‐type [wt] or mutant [mut]) were synthesized and cloned into a pGL‐3 luciferase control reporter vector (GenScript). BGC823 cells with miR‐92b mimics and NC and SGC7901 cells with miR‐92b inhibitor and NC were co‐transfected with the luciferase reporter vectors. A Dual‐Luciferase Reporter Assay System was employed to detect luciferase activity according to the manufacturer's instructions (Promega).

### 2.13 Transfection with the PI3K inhibitor ly294002 and Akt inhibitor MK2206

The PI3K inhibitor ly294002 and the Akt inhibitor MK2206 were purchased from Sigma‐Aldrich. Approximately 24 hours before transfection, miR‐92b mimics‐transfected BGC823 cells were plated in 6‐well plates at 35%‐55% confluence. Cells were then transfected with the PI3K inhibitor ly294002 or Akt inhibitor MK2206 at a working concentration of 50 nM or 5 µM, respectively, using Lipofectamine 2000 (Invitrogen) according to the manufacturer's protocol. After 48‐72 hours, the cells were collected for subsequent experiments.

### 2.14 Statistical analysis

Statistical analyses were performed with SPSS 19.0 and GraphPad Prism 5.0 software. Quantitative data are expressed as the mean ± SD. A two‐tailed Student's *t* test was employed to analyse differences between two groups. Multiple comparisons between groups were performed using analysis of variance (ANOVA) followed by a Student‐Newman‐Keuls test. Pearson's coefficient correlation or linear regression analysis was used to determine the association between two variables. Categorical data were evaluated using a chi‐square test. Survival rates were assessed using the Kaplan‐Meier method. A log‐rank test was used to compare significance. *P*‐values <0.05 were considered statistically significant. All experiments were performed at least in triplicate.

## 3 RESULTS

### 3.1 miR‐92b expression is upregulated and correlated with poor outcomes in GC patients

**Figure 1:**
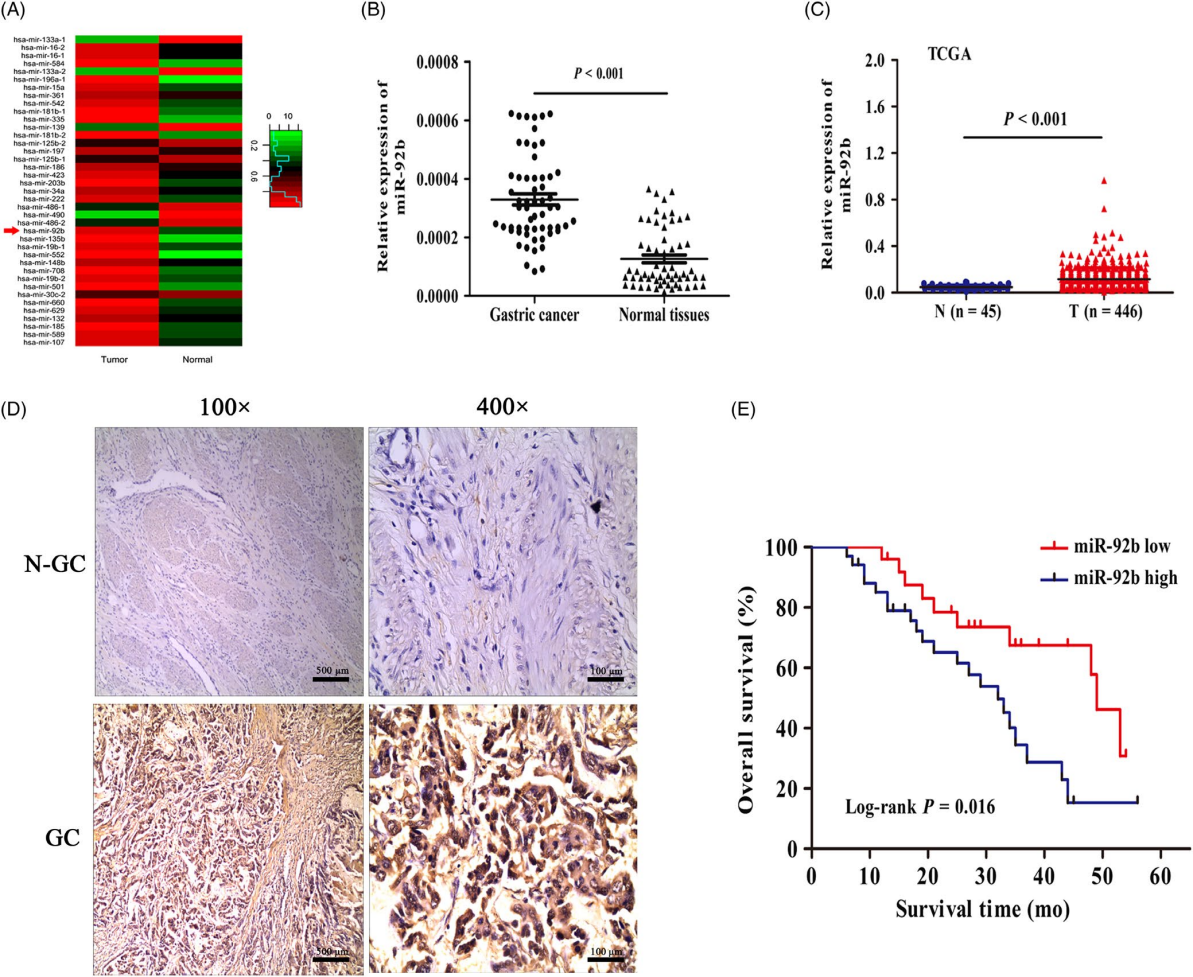
Clinical significance of miR‐92b in human gastric cancer (GC). A, Thirty‐nine miRNAs, which were available from TCGA database for miRNAs, were differentially expressed in normal (n = 45) and GC tumour (n = 446) samples. B, qRT‐PCR analysis of miR‐92b expression in 59 pairs of GC and peritumoural tissues. C, miR‐92b expression in GC tissues (n = 446) was higher than that in normal tissues (n = 45) (*P* < 0.001; TCGA). D, The expression status of miR‐92b in GC samples measured by in situ hybridization. Representative photomicrographs of miR‐92b expression in GC and peritumoural tissues. The positive brown signals show the relative expression level of miR‐92b. Original magnification, ×100, ×400. E, Kaplan‐Meier overall survival curves of 59 GC patients who were stratified on the basis of low expression (n = 25) and high expression (n = 34) of miR‐92b. miR‐92b upregulation was significantly associated with poor overall survival

To clarify the role of miR‐92b in GC progression, we first analysed the data, which represented GC (446 cases) and gastric (45 cases) tissues from the The Cancer Genome Atlas (TCGA) database, to identify miRNAs that are upregulated in GC tissues. Our results revealed that miR‐92b is one of the most markedly upregulated genes in GC tissues, and the expression level of miR‐92b was upregulated approximately 2.43‐fold (Figure 1A). Next, qRT‐PCR was performed to evaluate the expression of miR‐92b in 59 GC tissues and matched peritumoural tissues. The relative expression of miR‐92b in tumour tissues was significantly higher than in matched, peritumoural tissues (*P* < 0.001; Figure 1B). Our results were also verified by employing the TCGA database (Figure 1C). Moreover, ISH was employed to confirm the expression levels of miR‐92b in GC (Figure 1D). The results further indicated that miR‐92b expression was significantly upregulated in tumour cells. On the basis of ISH staining scores, 57.6% (34/59) of the GC samples exhibited positive miR‐92b expression in tumour cells; conversely, 32.2% (19/59) of the adjacent non‐tumour tissues showed positive miR‐92b expression (*P* = 0.006). Next, we studied the correlations between miR‐92b expression and clinicopathologic features of GC patients. As shown in Table 2, miR‐92b expression was significantly correlated with tumour size (*P* = 0.012) and differentiation (*P* = 0.040) but not with age, gender, lymph node metastasis or TNM stage. These data indicate that miR‐92b may play a crucial role in GC progression. Kaplan‐Meier curves and a log‐rank test indicated that miR‐92b overexpression was associated with poor outcomes in GC patients (log‐rank test, *P* = 0.016) (Figure 1E). In view of the above integrated analysis results, we hypothesize that increased expression of miR‐92b may promote GC progression.

**Table 2:** Expression of miR‐92b and DAB2IP in human gastric cancer according to clinicopathological characteristics of patients

Characteristics	Number	miR‐92b expression		*P* value	DAB2IP expression		*P* value
		High group	Low group		High group	Low group	
Total	59	34	25		23	36	
Gender							
Male	33	18	15	0.589	13	20	0.942
Female	26	16	10		10	16	
Age							
≤60	25	15	10	0.752	9	16	0.687
>60	34	19	15		14	20	
Tumour size							
<3 cm	18	6	12	0.012*	11	7	0.021*
≥3 cm	41	28	13		12	29	
Differentiation							
Low grade	35	24	11	0.040*	16	19	0.200
Middle and high grade	24	10	14		7	17	
Lymph node metastasis							
N_0_	28	12	16	0.065	15	13	0.091
N_1_	7	4	3		2	5	
N_2_ + N_3_	24	18	6		6	18	
Stages							
I	19	9	10	0.407	10	9	0.011*
II	13	7	6		8	5	
III + IV	27	18	9		5	22	

* *P* < 0.05 statistically significant difference.

### 3.2 Effect of miR‐92b on GC cell proliferation and apoptosis

**Figure 2:**
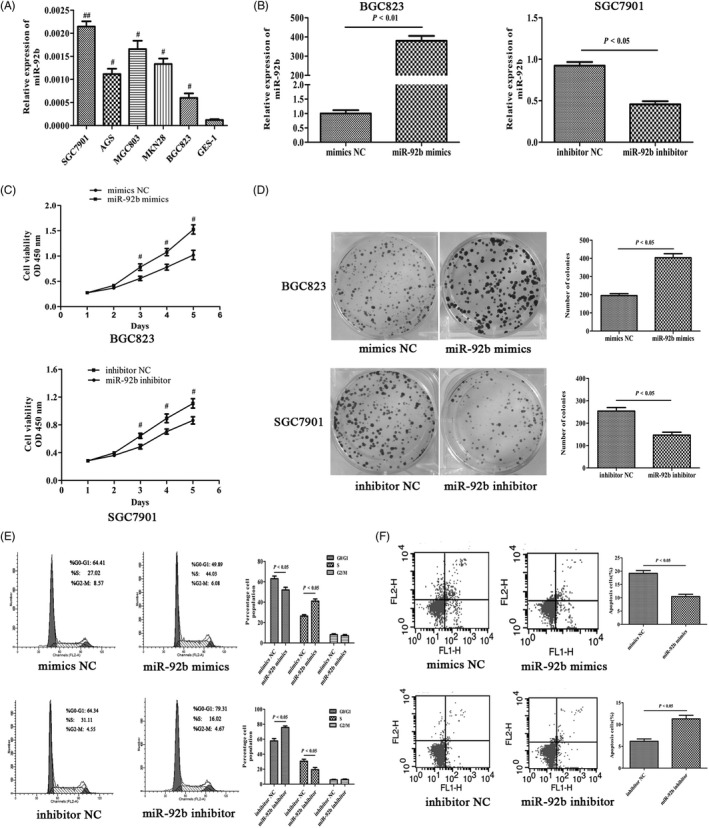
Effect of miR‐92b on gastric cancer (GC) cell proliferation and apoptosis. A, miR‐92b expression in the human epithelial cell line GES‐1 and in GC cell lines, including SGC7901, MGC803, AGS, MKN45 and BGC823. B, miR‐92b expression level in GC cell lines transfected with lentivirus miR‐92b mimics or miR‐92b inhibitor. C, Effects of miR‐92b on the proliferation of BGC823 and SGC7901 cell lines determined with a CCK‐8 assay. D, The proliferation of the transfected BGC823 and SGC7901 cells was detected with colony formation assays. E, The cell cycle distribution of the transfected BGC823 and SGC7901 cells was determined by flow cytometry. F, Apoptosis levels of the transfected BGC823 and SGC7901 cells were determined by flow cytometric analysis

Because miR‐92b was consistently upregulated in human GC specimens, we detected the expression level of miR‐92b in GC cell lines. The results revealed higher miR‐92b expression in SGC7901 and MGC803 cell lines than in BGC823 cells and the normal gastric epithelial cell line GES‐1 (Figure 2A). We transfected the high miR‐92b‐expressing cell line SGC7901 with a miR‐92b inhibitor and the low miR‐92b‐expressing cell line BGC823 with a miR‐92b mimics, and the transfection efficiencies of the SGC‐7901 and BGC823 cell lines were determined by qRT‐PCR (Figure 2B). The effects of miR‐92b on GC cell proliferation were assessed using CCK‐8, colony formation and FACS assays. As shown in Figure 2C, the proliferation rate was significantly increased in BGC823 mimics cells compared with control group cells. In contrast, the growth rate of SGC7901 inhibitor cells was much lower than that of the negative control cells. In addition, miR‐92b overexpression promoted the colony formation capability of BGC823 cells, while miR‐92b knock‐down attenuated the number of colonies (Figure 2D).

To explore the mechanism by which miR‐92b affects cell proliferation, we evaluated the effect of miR‐92b on cell cycle using flow cytometry. The results suggested that knock‐down of miR‐92b in SGC7901 cells led to an increase in the G_1_ phase population and a reduction in the S phase population, whereas overexpression of miR‐92b had the opposite effect (Figure 2E). These results demonstrate that miR‐92b functions as a G_1_‐S checkpoint to promote cell cycle progression in GC cells. These findings indicate that depletion of miR‐92b induced cell cycle arrest and prompted us to further explore the effect of miR‐92b on cell apoptosis. Flow cytometry analyses with annexin V and PI indicated that miR‐92b has an inhibitory role in cell apoptosis (Figure 2F).

### 3.3 DAB2IP is a direct target of miR‐92b

**Figure 3:**
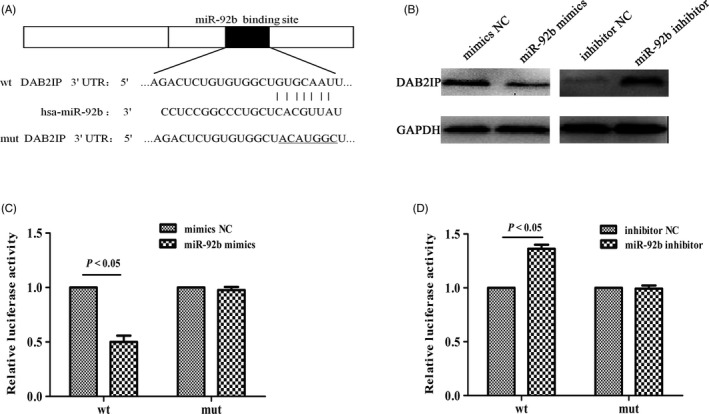
DAB2IP was a direct target of miR‐92b. A, The predicted binding site of miR‐92b in the 3’‐UTR of DAB2IP determined by TargetScan. B, Western blotting was employed to evaluate the effect of miR‐92b on the expression level of DAB2IP. C, D, Relative DAB2IP luciferase activity was evaluated in BGC823 cells co‐transfected with mimics or NC and pGL3‐DAB2IP or pGL3‐ DAB2IP‐mut and SGC7901 cells co‐transfected with inhibitor or NC and pGL3‐DAB2IP or pGL3‐ DAB2IP‐mut

Previous research has shown that miR‐92b may serve as a diagnostic biomarker, which led us to explore the underlying mechanism of miR‐92b overexpression.^14^, ^15^, ^28^ mRNA translation is primarily negatively regulated by miRNAs, and we hypothesized that the targets of miR‐92b are likely be tumour suppressors.^29^ To understand the regulation of miR‐92b in GC cells, three algorithms, miRanda (http://www.microrna.org/microrna/home.do), TargetScan (http://www.targetscan.org/) and PicTar (http://pictar.mdc-berlin.de/), were employed in combination with predict potential targets of miR‐92b, and DAB2IP was identified as a potential target (Figure 3A). DAB2IP silencing has been reported to be involved in gastrointestinal carcinogenesis and plays an important role in proliferation, migration and invasion.^30^, ^31^ As shown in Figure 3A, we found that miR‐92b can bind the 3′‐UTR of DAB2IP. Western blotting was employed to determine the change in DAB2IP expression in miR‐92b‐transfected GC cells (Figure 3B). Our results indicated that blocking of miR‐92b increased the expression of DAB2IP, while overexpression of miR‐92b downregulated the expression of DAB2IP. To further explore whether DAB2IP is a direct target of miR‐92b, a luciferase reporter assay was conducted. The wild‐type and a mutant DAB2IP 3’‐UTR containing putative target sites for miR‐92b were cloned into separate reporter plasmids (Figure 3C,D). The results demonstrated that miR‐92b mimics significantly reduced the activity of the luciferase reporter gene fused to the DAB2IP 3’‐UTR, whereas miR‐92b inhibitor induced an increase in luciferase activity. Mutation of the putative miR‐92b binding sites in the 3’‐UTR of DAB2IP did not affect the luciferase activity.

### 3.4 DAB2IP reversed the effects of miR‐92b mimics on GC cells

**Figure 4:**
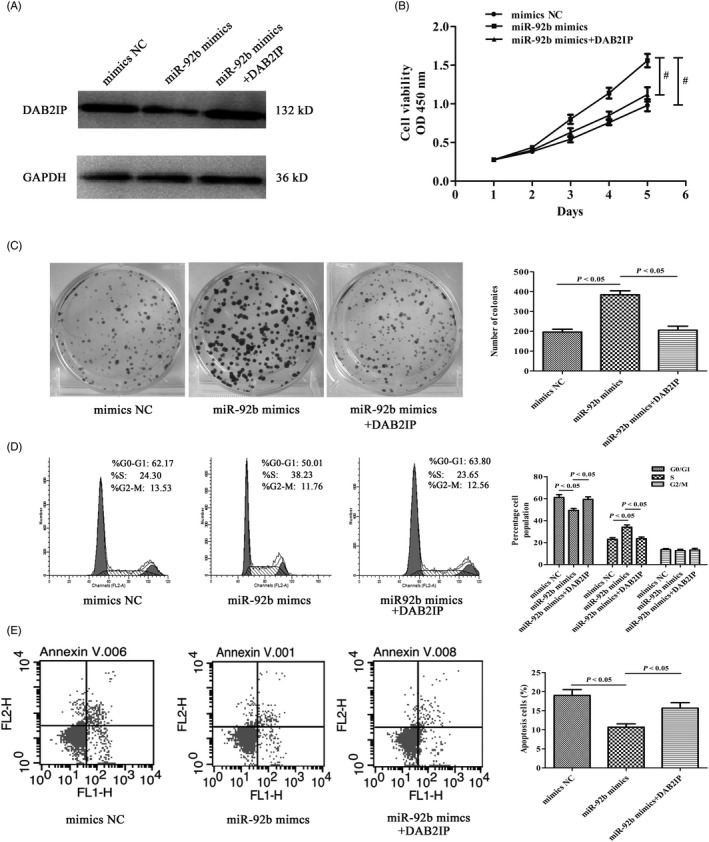
Overexpression of DAB2IP reversed the effect of miR‐92b in gastric cancer cells. A, The DAB2IP protein level was measured via Western blotting. B, DAB2IP reversed the effect of miR‐92b on cell growth determined with a CCK‐8 assay. C, Restoration of DAB2IP inhibited colony formation. D, Flow cytometry was conducted to confirm that DAB2IP could induce cell cycle arrest at G0/G1 phase. E, A flow cytometric assay was conducted to confirm that DAB2IP could reverse the effect of miR‐92b on cell apoptosis

First, the BGC823 mimics cell line was transfected with pcDNA3.1‐DAB2IP, and the transfection efficiency was confirmed by Western blotting. The results indicated that the expression of DAB2IP was increased (Figure 4A). To explore whether and how DAB2IP overexpression could reverse the effects of miR‐92b mimics, we conducted CCK‐8, colony formation and FACS assays. Figure 4B shows that the growth of BGC823 mimics cells transfected with pcDNA3.1‐DAB2IP was inhibited compared with negative control cells (*P* < 0.05). Colony formation assays indicated that the number of colonies decreased when DAB2IP was overexpressed (*P* < 0.05) (Figure 4C). Overexpression of DAB2IP also counteracted the effects of miR‐92b on cell cycle distribution base on flow cytometry analysis (Figure 4D). The reduced cell apoptotic indexes caused by miR‐92b were reversed by DAB2IP (Figure 4E). In conclusion, DAB2IP overexpression reversed the effects of miR‐92b on GC cell lines.

### 3.5 DAB2IP inhibitor reversed the effects of miR‐92b inhibitor on GC cells

**Figure 5:**
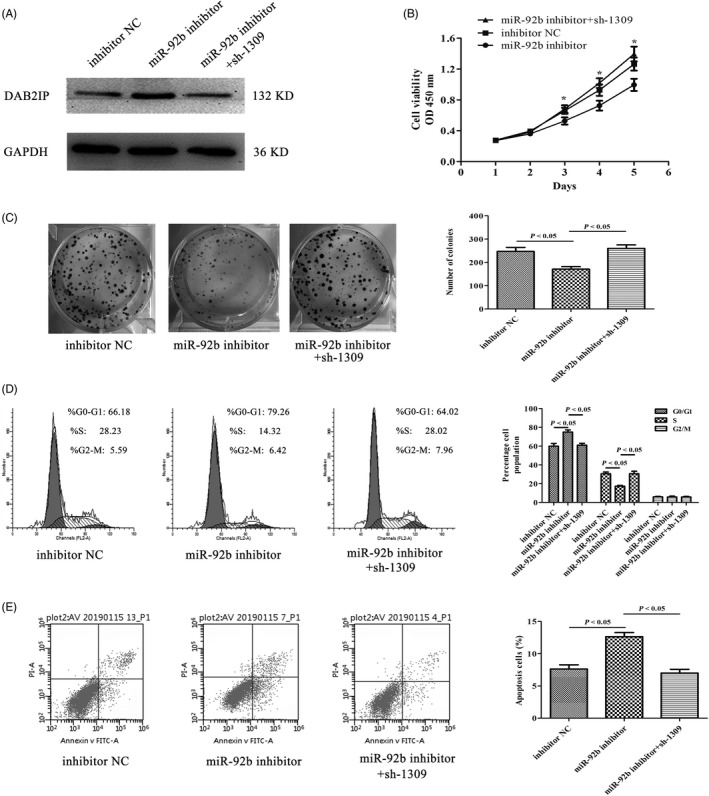
Downregulation of DAB2IP reversed the effect of miR‐92b inhibitor in gastric cancer cells. A, The DAB2IP protein level was measured via Western blotting. B, DAB2IP inhibitor reversed the effect of miR‐92b inhibitor on cell growth determined with a CCK‐8 assay. C, Downregulation of DAB2IP promoted colony formation. D, Flow cytometry was conducted to confirm that downregulation of DAB2IP could promote cycle transition from G0/G1 to S phase. E, A flow cytometric assay was conducted to confirm that downregulation of DAB2IP could reverse the effect of miR‐92b inhibitor on cell apoptosis

The SGC7901 miR‐92b inhibitor cell line was transfected with p‐SUPER‐sh‐1309, and the transfection efficiency was confirmed by Western blotting. The results indicated that the expression of DAB2IP was decreased in p‐SUPER‐sh‐1309‐transfected cell (Figure 5A). To explore whether and how DAB2IP downregulation could reverse the effects of miR‐92b inhibitor, we conducted CCK‐8, colony formation and FACS assays. Figure 5B shows that the cell growth of the SGC7901 miR‐92b inhibitor cell line transfected with p‐SUPER‐sh‐1309 was promoted compared with that of negative control cells (*P* < 0.05). Colony formation assays indicated that the number of colonies increased when DAB2IP was downregulated (*P* < 0.05) (Figure 5C). Downregulation of DAB2IP also reversed the effects of miR‐92b inhibitor on cell cycle distribution based on flow cytometry analysis (Figure 5D). The increased cell apoptotic indexes caused by miR‐92b inhibitor were reversed by DAB2IP inhibition (Figure 5E). In conclusion, DAB2IP downregulation reversed the effect of miR‐92b inhibitor on GC cell lines.

### 3.6 miR‐92b promoted GC cell tumorigenesis in vivo through downregulation of DAB2IP

**Figure 6:**
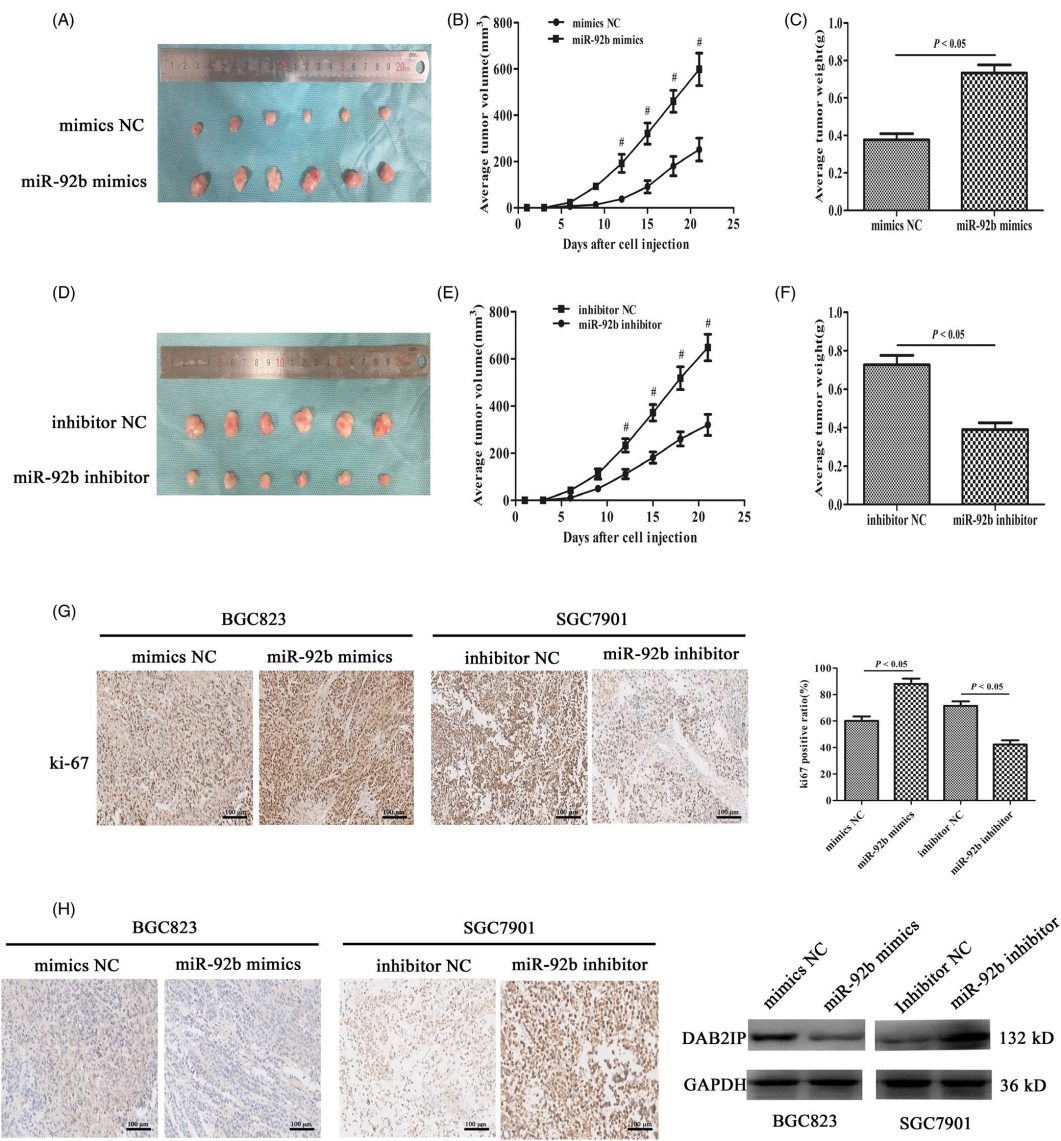
Ectopic expression of miR‐92b promoted tumour growth in vivo. A, D, Promotive effect of miR‐92b on tumour volume and weight in nude mice. B, E, The tumour size in nude mice from different groups was measured every 3 d after injection. C, F, The mice were euthanized, and the tumours were dissected for weighing after approximately 3 wk. G, The dissected tumours were analysed using a ki‐67 staining assay. H, The expression of DAB2IP was detected via immunohistochemical staining and Western blotting

SGC7901 inhibitor cells, control SGC7901 cells, BGC823 mimics cells and control BGC823 cells were injected into the left axilla of nude mice, and the volumes of the tumours were monitored every third day with a calliper. Our results indicated that the growth of miR‐92b‐overexpressing xenografts was markedly promoted in comparison with tumour growth in the negative control group (Figure 6A‐C). Conversely, the tumour volume of miR‐92b‐depleted xenografts was significantly less than that in the miR‐NC group (Figure 6D‐F). Consistent with the effects of different miR‐92b expression levels on cell proliferation in vitro, tumours from the mice injected with transfected cells displayed an identical trend based on Ki‐67 immunostaining (Figure 6G). The results indicated that miR‐92b promoted GC cell proliferation in nude mice. DAB2IP expression in transfected tumours was detected via IHC and Western blotting (Figure 6H). The results showed that the expression of DAB2IP was upregulated in the SGC7901 inhibitor group and downregulated in the BGC‐803 mimics group, which suggested that DAB2IP expression was negatively correlated with miR‐92b in vivo.

### 3.7 miR‐92b regulated the PI3K/AKT signalling pathway through DAB2IP

**Figure 7:**
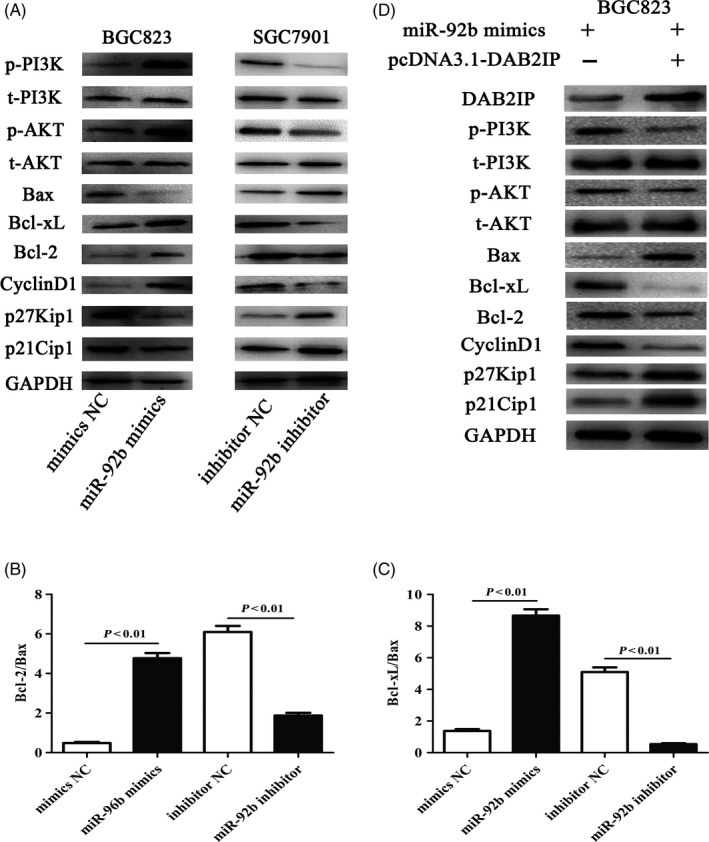
miR‐92b activated the PI3K/AKT signalling pathway via downregulation of DAB2IP. A, Inhibition of miR‐92b upregulated the protein levels of p21, p27 and Bax and downregulated the protein levels of p‐PI3K, p‐AKT, cyclin D1, Bcl‐2 and Bcl‐xl in SGC7901 inhibitor cells, and overexpression of miR‐92b upregulated the protein levels of p‐PI3K, p‐AKT, cyclin D1, Bcl‐2 and Bcl‐xl and downregulated the protein levels of p21, p27 and Bax in BGC823 mimics cells. Total PI3K and AKT levels were unchanged in the different groups. B, C, The ratios of Bcl‐2/Bax and Bcl‐xl/Bax were upregulated in BGC823 mimics cells and downregulated in SGC7901 inhibitor cells. D, Overexpression of DAB2IP downregulated the protein levels of p‐PI3K, p‐AKT, cyclin D1, Bcl‐2 and Bcl‐xl and upregulated the protein levels of p21, p27 and Bax. Total PI3K and AKT levels were unchanged in the different groups

Accumulated evidence indicates that the activation of the PI3K/AKT signalling pathway plays an indispensable role in downregulation of miR‐92b‐mediated nasopharyngeal and bladder cancer cell survival， and thus, we evaluated the correlation between miR‐92b and the PI3K/AKT signalling pathway in GC cells.^15^ Compared with control cells, the PI3K/AKT pathway was significantly activated, confirmed by the upregulated expression of both phosphorylated PI3K (p‐PI3K) and phosphorylated AKT (p‐AKT) levels in BGC823 mimics cell lines, while the expression levels of p‐PI3K and p‐AKT were downregulated in SGC7901 inhibitor cells (Figure 7A). Consistently, we also found that the G_1_‐S transition promoter cyclin D1 was upregulated in BGC823 mimics cell lines, whereas the protein expression levels of the G_1_ gatekeepers p21 and p27 were downregulated; in contrast, our results demonstrated that cyclin D1 was downregulated and p21 and p27 were upregulated in SGC7901 inhibitor cells (Figure 7A). The expression level of Bax (a characteristic pro‐apoptotic factor) was downregulated, but the Bcl‐2 and Bcl‐xl levels (indicating an anti‐apoptotic phenotype) were upregulated in BGC823 mimics cells; the opposite phenomenon was seen in SGC7901 inhibitor cells (Figure 7A). We also found that the ratios of Bcl‐2/Bax and Bcl‐xl/Bax were upregulated in BGC823 mimics cells and downregulated in SGC7901 inhibitor cells (Figure 7B,C). The BGC823 mimics cell line was transfected with pcDNA3.1‐DAB2IP to restore DAB2IP to further illustrate the function of DAB2IP in miR‐92b‐mediated GC progression via the PI3K/AKT signalling pathway (Figure 7D). Our results showed that the protein expression of DAB2IP was restored, and we found that the restoration of DAB2IP increased the expression levels of P21, P27 and Bax and decreased the expression of cyclin D1, Bcl‐2 and Bcl‐xl.

### 3.8 Inhibition of the PI3K/AKT signalling pathway attenuated the proliferation induced by miR‐92b

**Figure 8:**
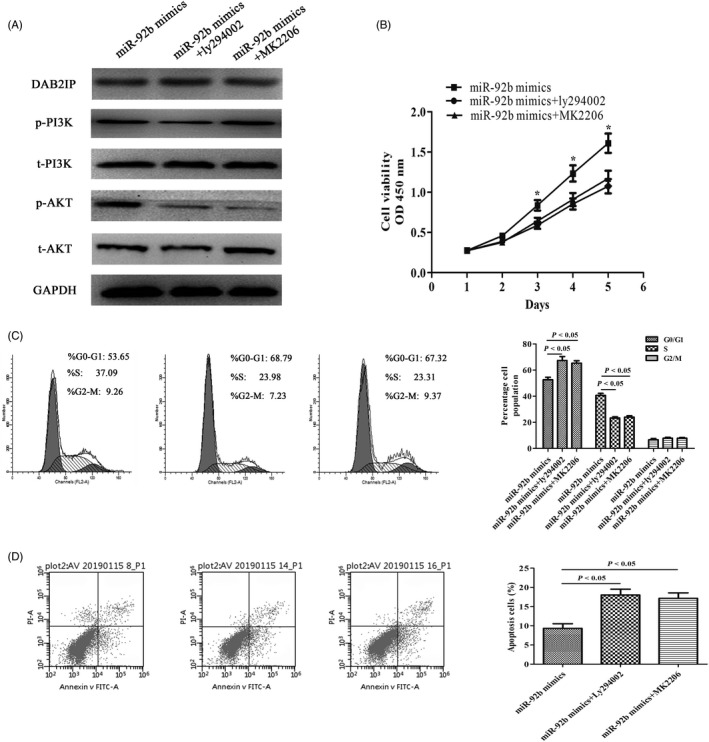
Inhibition of PI3K/AKT signalling pathway attenuated the proliferation induced by miR‐92b. A, The PI3K inhibitor ly294002 and the AKT inhibitor MK2206 suppressed the protein levels of p‐PI3K and p‐AKT. B, The PI3K inhibitor ly294002 and the Akt inhibitor MK2206 reversed the effect of miR‐92b mimics on cell growth determined with a CCK‐8 assay. C, Flow cytometry was conducted to confirm that a PI3K/AKT signalling pathway inhibitor could induce cell cycle arrest at G0/G1 phase. D, A flow cytometric assay was conducted to confirm that a PI3K/AKT signalling pathway inhibitor could reverse the effect of miR‐92b on cell apoptosis

We used the specific inhibitor of PI3K ly294002 to inhibit the expression of PI3K and found that the expression levels of phosphorylated PI3K and phosphorylated AKT were reduced in miR‐92b mimics‐transfected cells. Moreover, we used the specific inhibitor of AKT MK2206 to suppress the expression of AKT and found that the expression of phosphorylated AKT was reduced in miR‐92b mimics‐transfected cells (Figure 8A). To explore whether the PI3K/AKT signalling pathway can attenuate the proliferation induced by miR‐92b, we conducted CCK‐8 and FACS assays. Figure 8B shows that the cell growth of BGC823 mimics cells transfected with ly294002 or MK2206 was inhibited compared with that of negative control cells (*P* < 0.05). Transfection with ly294002 or MK2206 also reversed the effects of miR‐92b mimics on cell cycle distribution based on flow cytometry results (Figure 8C). The reduced cell apoptotic indexes caused by miR‐92b mimics were reversed by ly294002 or MK2206 (Figure 8D). In conclusion, the PI3K/AKT signalling pathway inhibitor attenuated the proliferation induced by miR‐92b.

### 3.9 Clinical significance of miR‐92b and DAB2IP expression in GC

**Figure 9:**
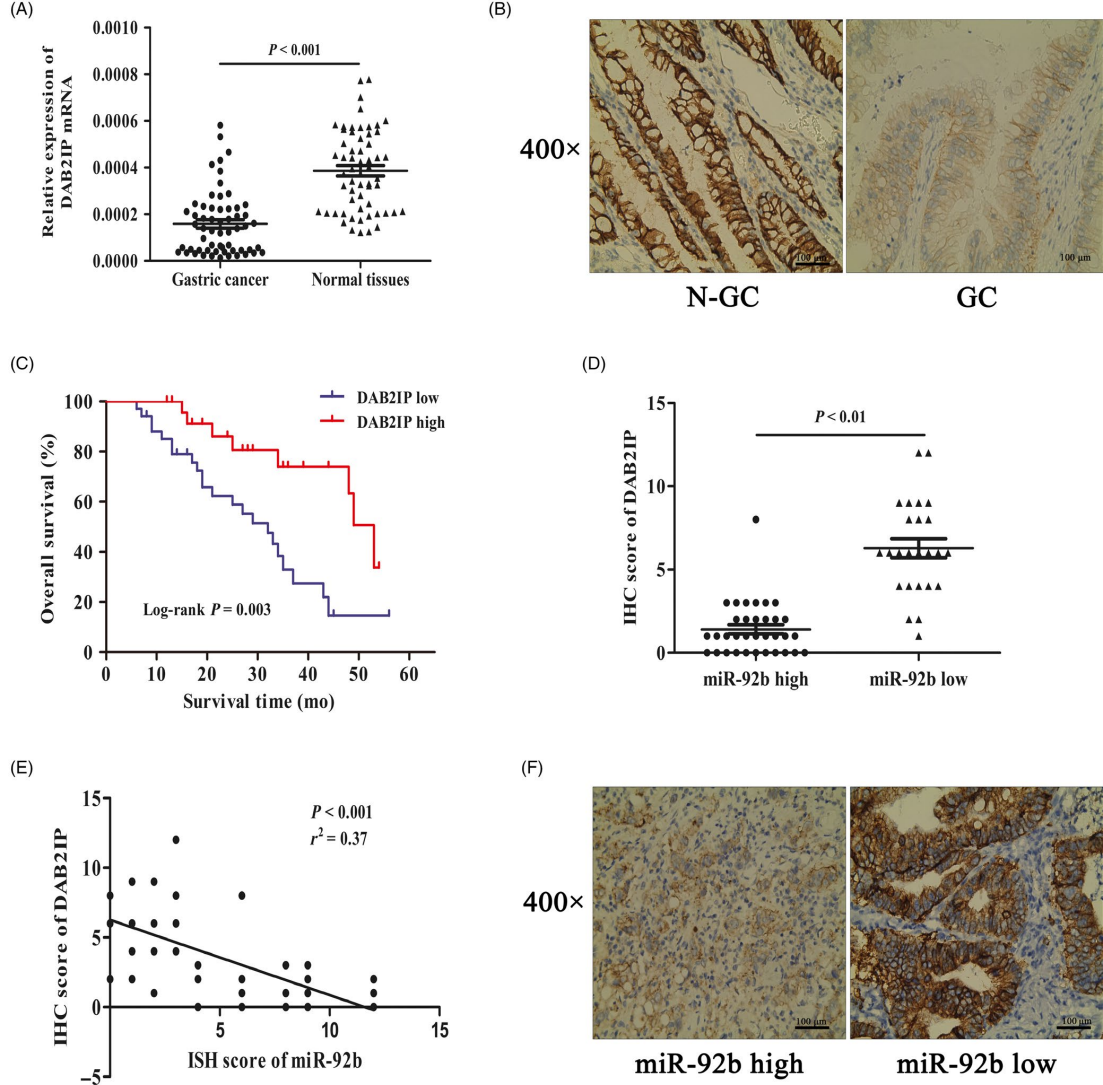
The relationships between miR‐92b expression and the expression of DAB2IP in human gastric cancer (GC) tissues. A, qRT‐PCR analysis of DAB2IP expression in 59 pairs of GC and peritumoural tissues. B, The expression status of DAB2IP in GC samples measured via immunohistochemical staining (IHC). Representative photomicrographs of DAB2IP expression (IHC) in GC and peritumoural tissues. Original magnification, ×400. C, Kaplan‐Meier overall survival curves of 59 GC patients who were stratified on the basis of low expression (n = 36) and high expression (n = 23) of DAB2IP. DAB2IP downregulation was significantly associated with poor overall survival. D, GC patients with high miR‐92b expression had significantly lower DAB2IP expression than those with low miR‐92b expression. E, miR‐92b was inversely correlated with DAB2IP (*r* ^2^ = 0.37, *P* < 0.001). F, Representative photomicrographs of DAB2IP expression (IHC) in GC tissues with high and low miR‐92b expression

To further confirm the expression levels of DAB2IP in tumour cells, qRT‐PCR and IHC analyses of the GC samples obtained from 59 GC patients were performed. Our results indicated that the mRNA and protein expression of DAB2IP was significantly lower in GC tissues than in peritumoural tissues, and we found that DAB2IP was predominantly expressed in the cytoplasm of both GC and normal mucosal cells (Figure 9A,B). On the basis of IHC staining scoring, the DAB2IP was positively expressed in 39.0% (23/59) of GC samples; in contrast, 69.5% (41/59) of the matched tumour‐adjacent tissues exhibited DAB2IP‐positive expression (*P* = 0.01). Next, we investigated correlations between DAB2IP expression and clinicopathologic parameters in GC patients. As shown in Table 2, DAB2IP expression was significantly associated with tumour size (*P* = 0.021) and TNM stage (*P* = 0.011). More intriguingly, Kaplan‐Meier analysis indicated that the presence of DAB2IP‐positive tumours was significantly correlated with better overall survival of patients (*P* = 0.003) (Figure 9C). The correlation between miR‐92b and DAB2IP was investigated based on the available miR‐92b ISH data and DAB2IP IHC data, and patients with higher miR‐92b expression were found to have markedly lower DAB2IP expression than those with lower miR‐92b expression (Figure 9D). Notably, we also found that DAB2IP expression was inversely associated with miR‐92b expression (*r* ^2^ = 0.37, *P* < 0.001, Figure 9E). The results further support an inverse association between miR‐92b and DAB2IP expression in human GC tissues (Figure 9F). Overall, DAB2IP is downregulated in human GC, and miR‐92b is reversely correlated with DAB2IP expression in GC tissues.

## 4 DISCUSSION

Successive accumulation of genetic alterations, such as amplification or mutation of oncogenes and loss of function or mutation of tumour suppressor genes, is the main reason for the carcinogenesis of GC.^32^ Despite the extensive information about GC at the genetic and molecular levels that has been gradually gathered, most patients are diagnosed at advanced stages and lose the opportunity for surgical therapy due to differing clinical courses and specific biomarkers, which has compelled researchers to explore the molecular mechanisms of GC progression and to identify effective therapeutic strategies for treatment of this fatal disease.

MicroRNAs play a significant role in a wide range of biological events, such as tumorigenesis and are known to function as regulators in GC cell proliferation.^33^, ^34^ We conducted an integrated analysis and then confirmed miRNAs with altered expression levels in GC cells by employing RNA‐Seq data (from TCGA), and we confirmed that miR‐92b is a clinically extraordinary miRNA in GC. By combining TCGA, qRT‐PCR and ISH data, we found that miR‐92b was highly expressed in GC tissues in comparison with expression in adjacent non‐tumour tissues, suggesting that miR‐92b might be involved in GC development. Moreover, ISH analysis indicated that overexpression of miR‐92b was markedly correlated with tumour size, tumour differentiation and poor overall survival of GC patients. miR‐92b was also highly expressed in GC cell lines but weakly expressed in the GES‐1 cell line. Because the GC tissues and GC cell lines had high miR‐92b expression levels, we posed a hypothesis that miR‐92b may function as an oncogene and play important crucial roles in GC cell progression. As we predicted, the tumorigenic ability of miR‐92b assessed with CCK‐8, colony formation and flow cytometric assays was significantly promoted, suggesting a role of miR‐92b in cancer cell proliferation. Experiments in vivo also demonstrated that overexpression of miR‐92b was positively associated with larger xenografts in a nude mouse model constructed by injection of transfected GC cells. Furthermore, IHC analysis indicated that overexpression of miR‐92b accelerated tumour growth and inhibited cell apoptosis, which was evidenced by strong Ki‐67 staining.

To further explore the detailed mechanism of the biological function that miR‐92b targeted, several biological software programmes were employed to predict the possible targets of miR‐92b, and we identified DAB2IP as a possible candidate target. DAB2IP, a member of the RAS‐GAP protein family, is known to serve as apoptosis signal‐regulating kinase 1‐interacting protein‐1 (AIP1) and is a putative tumour suppressor in various cancers by regulating multiple signalling pathways, including PI3K‐AKT, Ras‐ERK and ASK1‐JNK pathways.^35^ Accumulated evidence has shown that DAB2IP is inactivated and functions as a tumour suppressor in GC.^36^, ^37^ Further studies have indicated that loss of DAB2IP is involved in driving tumour cell proliferation, initiating epithelial‐mesenchymal transition and metastasis, acquisition of radio‐or chemotherapy resistance and inhibition of tumour cell apoptosis.^16^, ^17^, ^38^, ^39^ A luciferase reporter assay and Western blotting were employed to verify the regulatory relationship between miR‐92b and DAB2IP in vitro*,* and in vivo*,* the expression level of DAB2IP in tumours collected from the tumour xenograft assay further demonstrated that DAB2IP was a direct target of miR‐92b. To explore whether the effect of miR‐92b on GC cell biological functions was reversed by DAB2IP, we transfected the BGC823 mimics cell line with pcDNA3.1‐DAB2IP. Our results indicated that DAB2IP is a direct target of miR‐92b, evidenced by inhibition of cell growth, a decrease in the number of colonies, cell cycle arrest at G_0_/G_1_ phase and acceleration of cell apoptosis.

PI3K is involved in the regulation of diverse cellular processes, such as cell proliferation, motility, apoptosis, transcription and angiogenesis.^40^, ^41^ AKT, the main downstream effector of PI3K, is subsequently activated by PI3K activation and phosphorylates multiple enzymes, kinases and transcription factors to regulate various biological processes.^42^ DAB2IP has been reported to suppress the PI3K‐AKT pathway, leading to reduced cell proliferation and increased cell apoptosis.^17^ Moreover, CHIP controls glioma proliferation and growth through PTEN/PI3K/AKT signalling via upregulation of miR‐92b.^43^ However, the correlation among miR‐92b, DAB2IP and PI3K/AKT signalling in GC remains unknown. We hypothesized that miR‐92b activates the PI3K/AKT signalling pathway via loss of DAB2IP in GC. In the present study, we found that miR‐92b is critical for GC progression via the PI3K/AKT signalling pathway, evidenced by the increased protein levels of phosphorylated PI3K and AKT. Our results suggest that the PI3K/AKT signalling pathway participates in miR‐92b‐mediated cell progression in GC. To verify the effect of DAB2IP on the PI3K/AKT signalling pathway in GC, Western blotting analysis of BGC823 cells co‐transfected with miR‐92b mimics and pcDNA3.1‐DAB2IP was performed. Our results suggest that DAB2IP can inhibit the PI3K/AKT signalling pathway activated by miR‐92b.

Malignant proliferation and apoptosis inhibition are two of the most malignant GC phenotypes.^20^ Cell proliferation is tightly correlated with the regulation of cell cycle progression.^44^ This prompted us to investigate the relationship between miR‐92b expression and cell cycle progression in GC. Our previous results indicated that miR‐92b promotes GC cells from G0/G1 phase into S phase, with a concomitant increment in cell growth compared with the control group. An increasing number of studies have reported that the regulation of G_1_/S phase transition abnormally occurs in tumour progression and is associated with changes in CDK inhibitors or cyclins.^45^, ^46^ p21 and p27, which are cyclin‐dependent kinase inhibitors, induce cell cycle arrest in response to multiple stimuli, and cyclin‐D1 is the major cyclin regulating cell cycle transition from G0/G1 phase to S phase of the cell cycle.^47^, ^48^ Thus, the expression levels of PI3K/AKT pathway target genes, such as cyclin‐D1, p21 and p27, which are important components and key links in cell proliferation, were determined by Western blotting. We found that G_1_/S phase transition induced by miR‐92b was associated with upregulation of cyclin‐D1 and downregulation of p21 and p27. The results showed that miR‐92b plays a substantial role in cell cycle progression. Concomitant with the facilitation of cell proliferation, the growth facilitative effect of miR‐92b was also related to a reduction in apoptosis, as shown by flow cytometric analysis of the miR‐92b‐expressing cultured GC cell lines and the protein expression levels of a series of apoptosis‐related genes. We found that miR‐92b affects apoptosis of GC cells. Our data showed that there was a significant decrease in apoptotic indexes in BGC mimics cell lines and a marked increase in apoptotic indexes in SGC7901 inhibitor cell lines. The effect of miR‐92b on cell apoptosis was correlated with alterations in apoptosis‐related genes, such as Bax, Bcl‐2 and Bcl‐xl, which are also PI3K/AKT pathway‐regulated gene products.^49^ Our results indicated that miR‐92b significantly decreases the protein level of Bax and increases the protein level of Bcl‐2 and Bcl‐xl. Therefore, miR‐92b exerts its effect by regulating cell cycle and cell survival to promote tumour growth.

We further confirmed the clinical relevance of miR‐92b and DAB2IP expression in GC. DAB2IP mRNA and protein expression levels were detected in 59 pairs of GC tissues from patients, and we found that DAB2IP expression was significantly downregulated in GC. Our results indicated that low DAB2IP expression was significantly associated with tumour size, TNM stage and a shorter overall survival time. We also found that tumours with high miR‐92b status had a significantly lower DAB2IP IHC score than tumours with low miR‐92b status, indicating that miR‐92b expression was inversely correlated with DAB2IP expression.

Collectively, for the first time, this study identified the DAB2IP gene as a target of miR‐92b, which reveals its role in GC development. miR‐92b substantially promoted GC cell proliferation by inhibiting the expression of DAB2IP. Considering the proliferative effects of miR‐92b in tumour cells, miR‐92b may be a potential diagnostic and therapeutic agent for GC.

## CONFLICT OF INTEREST

The authors declare no conflict of interest.
